# Analysis of Local Free Volume and Hydration in Pharmaceutical Polymer Films Using Positron Annihilation Lifetime Spectroscopy

**DOI:** 10.3390/ijms27219518

**Published:** 2026-10-24

**Authors:** Mariam Majida Shokoya, Károly Süvegh, Zsófia Vilimi, Nikolett Kállai-Szabó, Romána Zelkó, István Sebe

**Affiliations:** 1 University Pharmacy Department of Pharmacy Administration, Semmelweis University, 7-9 Hőgyes Street, 1092 Budapest, Hungary; shokoya.mariam.majida@gmail.com (M.M.S.); sebe.istvan@semmelweis.hu (I.S.); 2 Center for Pharmacology and Drug Research & Development, Semmelweis University, 1085 Budapest, Hungary; vilimi.zsofia@semmelweis.hu (Z.V.); kallai.nikolett@semmelweis.hu (N.K.-S.); 3 Department of Nuclear Chemistry, Institute of Chemistry, Faculty of Science, Eötvös Loránd University, 1518 Budapest, Hungary; karoly.suvegh@ttk.elte.hu; 4 Department of Pharmaceutics, Faculty of Pharmaceutical Sciences, Semmelweis University, 7-9 Hőgyes Street, 1092 Budapest, Hungary

**Keywords:** positron annihilation lifetime spectroscopy, ortho-positronium lifetime, free volume hole size, pharmaceutical film-forming polymers, poly(vinyl alcohol), hydroxypropyl cellulose, sodium alginate, residual moisture content, equilibrium swelling index, quality by design

## Abstract

The relationship between the nanoscale free volume of pharmaceutical polymer films and their hydration behavior remains poorly understood. Eight pharmaceutical polymers were screened at three concentration levels within a quality-by-design framework; only two poly(vinyl alcohol) grades (PVA EG-30P and EG-40P), hydroxypropyl cellulose (HPC), and sodium alginate (Na-alginate) formed continuous films. These were characterized by positron annihilation lifetime spectroscopy (PALS), loss on drying (LOD), and swelling analysis. PALS clearly differentiated HPC, Na-alginate, and PVA, whereas the two PVA grades were similar and the concentration had only a minor effect. HPC showed the largest free volume holes (86.7–89.5 Å^3^), and Na-alginate the smallest holes (36.9–39.6 Å^3^) together with the highest LOD (20.2–21.6%). Across the 12 polymer–concentration combinations, the free volume hole volume correlated inversely with LOD (Spearman ρ = −0.85) but not with the equilibrium swelling index, as both HPC and Na-alginate swelled considerably more than PVA. Thus, PALS discriminates film-forming pharmaceutical polymers and reflects their residual water content, but the free volume of the air-dried films does not predict their swelling capacity.

## 1. Introduction

The organization and interactions of polymer chains, together with the amount of bound water present in the system, govern the interaction of a polymer matrix with water and thereby the properties of hydrated polymer thin films. These factors determine the local free volume available for swelling, the amount of fluid taken up during hydration, and the resulting dimensional changes. A comprehensive understanding of such systems at both the microstructural and molecular level is therefore essential for the rational selection and optimization of pharmaceutical and biomaterial polymers for a given application. Conventional macroscopic measurements, however, provide only limited insight into the microstructural mechanisms underlying the macroscopically observed water uptake, swelling, and dimensional changes.

Positron annihilation lifetime spectroscopy (PALS) is a powerful technique for probing free volume characteristics in polymeric materials at the nanoscale level [1,2]. In the PALS lifetime spectrum, the ortho-positronium (o-Ps) component, associated with the longest lifetime, is generally characterized by two parameters: its lifetime (τ_3_) and relative intensity (I_3_). By applying the Tao–Eldrup model to the o-Ps lifetime component, the mean radius and the corresponding volume of free volume cavities can be estimated, while the o-Ps intensity provides qualitative information about the relative concentration of the cavities within the polymer matrix [3,4].

The PALS technique provides insight into several aspects of the microstructural organization of polymer matrices, particularly the size, distribution, and connectivity of free volume elements. The chemical nature of the polymer, such as the presence of side chains, the rigidity of polymer chains, the density of crosslinks, and the degree of crystallinity, or amorphous nature all significantly influence the size and distribution of free volume in a polymer matrix [5,6,7]. Interpreting the PALS-derived parameters together with the loss-on-drying (LOD) and swelling data therefore allows the comparison of polymer matrices across microstructural and macroscopic levels, and may provide insight into the molecular-level mechanisms underlying their hydration and swelling behavior.

A structurally diverse yet functionally relevant set of eight hydrophilic polymers, which are commonly used in pharmaceutical applications, was selected for the initial screening in this study: two PVA grades, Poloxamer, two Carbomer grades, HPC, Na-CMC, and Na-alginate. The selected polymers comprise natural, semi-synthetic, and synthetic materials of either a nonionic or anionic character, with distinct functional groups that influence their interactions with water. Na-alginate and Na-CMC are anionic linear polysaccharides that contain carboxylate functional groups, which promote hydration and render their aqueous behavior sensitive to ionic conditions [8,9,10]. PVA and HPC are nonionic polymers with an extensive hydrogen-bonding capacity. PVA is a semicrystalline polymer, rich in hydroxyl groups, whereas HPC is a hydroxypropyl-substituted cellulose ether [11,12,13,14]. Poloxamer is a triblock copolymer, consisting of PEO-PPO-PEO triblocks. It possesses thermoreversible gelation, which is temperature- and concentration-dependent, and, owing to its amphiphilic nature, it can self-assemble into micelles in aqueous solutions [15,16]. Carbomer is a synthetic, crosslinked poly(acrylic acid) network, whose water uptake and swelling are strongly pH-dependent because of the ionization of its carboxylic acid groups [17].

The investigated polymers thus differ in polymer-chain architecture, functional group chemistry, ionization behavior, and intermolecular organization. Consequently, they are expected to exhibit distinct polymer–water interactions, hydration characteristics, and swelling behavior. To complement the comparison of chemically distinct polymer types, two PVA grades (PVA EG-30P and PVA EG-40P) and two Carbomer grades (Carbomer 971 and Carbomer 974) were included to assess grade-dependent differences in rheological and film-forming properties, as well as in PALS-derived parameters and swelling properties for the film-forming grades. If such differences are observed, they may reflect grade-dependent variations in molecular characteristics, including molecular weight, solution viscosity, and network organization. Each polymer was screened at three concentration levels. Collectively, the selected polymer set provides a basis for systematically evaluating the effects of polymer type, polymer grade, and concentration on film formation, and on free volume-related microstructural parameters, LOD, and swelling behavior in the resulting thin films.

The polymer characterization strategy was structured within a quality-by-design (QbD) framework and was aligned with the identified critical material attributes (CMAs) and critical process parameters (CPPs), and systematic risk assessment. In the present study, QbD served as a prospective framework for structuring the analyses and for identifying the factors that may influence thin film formation and film properties. It was not intended to establish a pharmaceutical design space or to conclude therapeutic performance. Initially, all eight polymers were screened at three concentration levels for apparent viscosity and film-forming capacity. Because only four of them (the two PVA grades, HPC, and Na-alginate) formed continuous films under the applied conditions (Section 2.2), a thin film thickness measurement, LOD, PALS, and swelling analysis were performed only on these four film-forming polymers, at three concentration levels.

Although PALS has been applied to a wide range of pharmaceutical excipients, drug delivery systems, and medical devices, few studies have systematically compared chemically distinct film-forming pharmaceutical polymers prepared under identical conditions while directly relating PALS-derived free volume descriptors to the residual moisture content and swelling behavior of the resulting films [1]. We hypothesized that polymer-specific differences in local free-volume descriptors are associated with distinct hydration and swelling behavior. Accordingly, the aims of this work were (i) to investigate whether PALS is suitable for differentiating pharmaceutical polymers at the microstructural level, (ii) to relate PALS-derived parameters to macroscopic hydration behavior in terms of the underlying molecular features, including interchain interactions, crystallinity, and polymer chain mobility, and (iii) to assess whether the free volume of the air-dried films is related to their residual moisture content and whether it can predict their swelling capacity. The investigated systems did not contain an active pharmaceutical ingredient (API), nor additional formulation-specific excipients. Consequently, the API loading capacity, drug-release performance, mechanical performance, and therapeutic efficacy were outside the scope of this study.

## 2. Results and Discussion

### 2.1. QbD-Based Polymer Screening and Risk Assessment

A preliminary QbD- and risk-based screening framework was used to identify and prioritize the polymers and the processing variables with potential relevance to thin film formation and physicochemical behavior, and to structure the physicochemical characterization of the drug-free polymeric thin film matrices.

**Figure 1:**
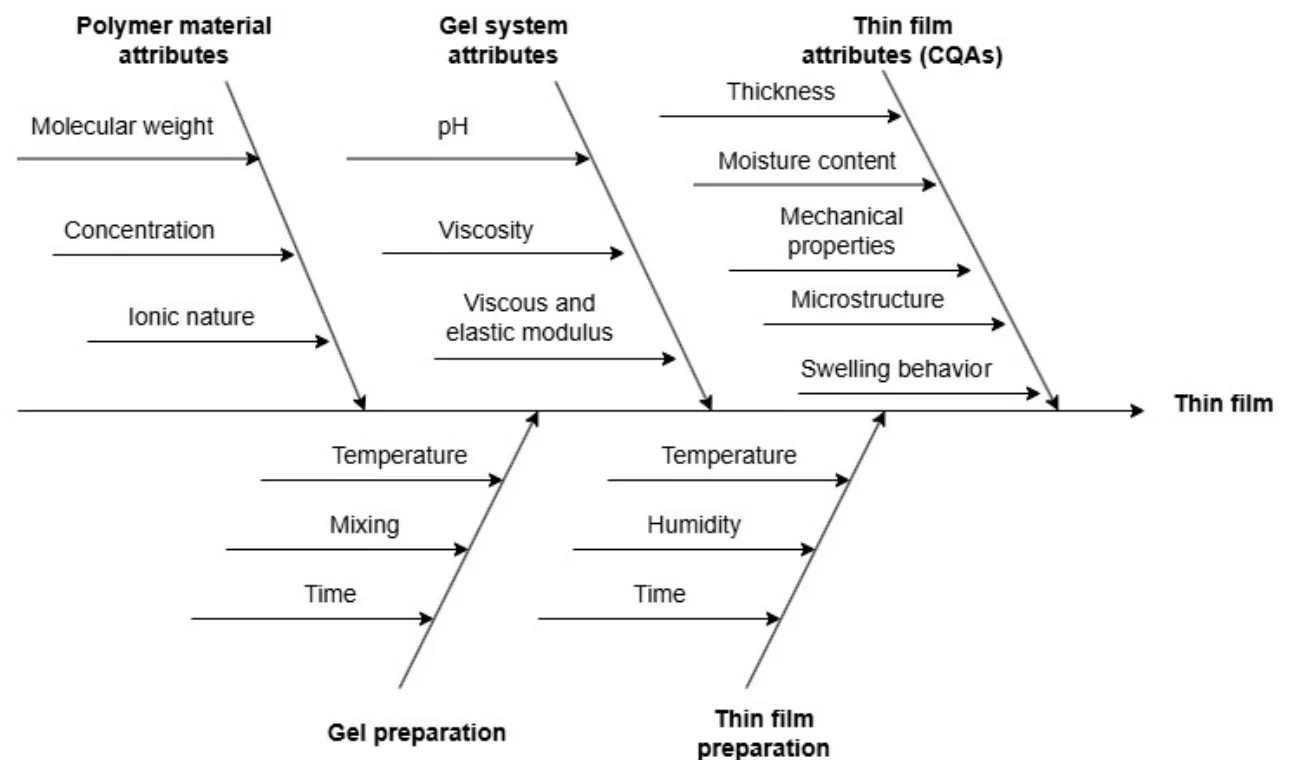
Ishikawa diagram illustrating polymer- and technology-related variables and their potential effects on the critical quality attributes of drug-free polymeric thin films.

Figure 1 presents the Ishikawa diagram of the relevant CMAs and CPPs, summarizing their potential effects on the physicochemical properties of the thin films during polymer selection, and gel and thin film preparation. Because the present work examined drug-free polymer films without formulation-specific excipients, the scope was limited to the physicochemical and microstructural characteristics of the polymer matrices. The Ishikawa diagram illustrates the potential relationships between the properties of the polymeric raw materials, processing parameters, and the attributes of the resulting gels and API-free thin films. The identified critical quality attributes (CQAs) include the thin film thickness, moisture content, mechanical properties, microstructure, and swelling behavior. Overall, the diagram provides a QbD-informed framework for organizing the potential sources of variability and their combined effects on thin film matrices.

Based on the factors identified in the Ishikawa diagram, a preliminary failure mode and effects analysis (FMEA)-based risk assessment was performed to prioritize the potential impact of CMAs and CPPs on the API-free thin films (Table 1). Each factor was scored for severity (S), occurrence (O), and detectability (D) on a scale of 1–5, and the risk priority number (RPN = S × O × D) was calculated [18,19,20]. The RPN was used to select the factors for further experimental investigation. In addition, the scores were categorized on a three-level scale (High, Medium, or Low) according to their severity and potential impact on the thin film physicochemical properties [18,21]. The ‘✓’ symbol indicates that the given CMA or CPP has a relevant impact on the corresponding CQA, whereas blank cells denote a negligible effect.

Risk assessment was performed by evaluating the overall influence of each CMA and CPP across all associated CQAs. The resulting scores were categorized according to predefined thresholds: values > 60 were classified as high risk, values between 30 and 59 as medium risk, and values < 29 as low risk [19]. This assessment prioritized the polymer type, apparent viscosity, and process parameters of thin film preparation affecting API-free thin film CQAs.

The CQAs of the thin films were subsequently characterized through a series of analytical investigations, in which the gel viscosity, film-forming capacity, thin film thickness, LOD, microstructure, and swelling behavior were evaluated. Mechanical properties were included in the Ishikawa diagram, since they are considered CQAs and are influenced by the polymer microstructure and hydration-induced swelling. However, owing to the specific focus of the present study on the relationship between local free volume characteristics and hydration behavior, mechanical properties were not evaluated experimentally.

### 2.2. Film-Forming Assessment

The film-forming capacity of the polymers was evaluated under the applied preparation, casting, and drying conditions. The formation of a continuous and uniform thin film was used as an initial selection criterion for the subsequent physicochemical characterization (film thickness, LOD, PALS, and swelling measurements). Polymer classification based on film-forming outcome, ionic nature, origin, and structural features relevant to screening are summarized in Table 2.

Among the eight polymers screened, PVA EG-30P, PVA EG-40P, HPC, and Na-alginate formed continuous films and were therefore evaluated in the subsequent thin film analysis. Carbomer 971, Carbomer 974, Poloxamer, and Na-CMC did not form continuous films under the tested conditions.

The film-forming outcome can be explained by the mechanism of film formation from aqueous polymer systems. During water evaporation, the polymer chains are brought into close contact, interpenetrate, and entangle, and a continuous film is obtained only if the resulting network is sufficiently cohesive to withstand the shrinkage stresses that develop during drying [22]. Linear, high-molecular-weight polymers with numerous hydrogen-bonding groups, such as PVA, HPC, and Na-alginate, typically fulfill these requirements. In contrast, Carbomers are covalently crosslinked poly(acrylic acid) polymers which, in neutralized aqueous gels, are present as individually swollen microgel particles rather than as freely interpenetrating chains [17,23]. Upon drying, the crosslinked microgel structure may restrict chain interdiffusion between neighboring particles, thereby hindering their coalescence into a cohesive, continuous matrix. Poloxamer 407 is a relatively low-molecular-weight (approximately 12.6 kDa) amphiphilic triblock copolymer. Its gel state arises primarily from the temperature- and concentration-dependent packing and interactions of self-assembled micelles rather than the formation of an entangled polymer network [15,16,24]. This micellar structure is lost upon water removal, and the relatively short chains and the crystallization of the PEO blocks prevent the formation of a cohesive, continuous film. Na-CMC is generally considered a film-forming polymer; therefore, its failure to form continuous films is attributed to the applied processing window rather than to its chain architecture. Owing to the high viscosity of the 7H4F grade, the processable concentrations were limited to 1.13–2.38 *w*/*w*% (Section 3.2). In the absence of a plasticizer, Na-CMC, an anionic polyelectrolyte with semiflexible chains, may form stiff, brittle layers that are susceptible to cracking under drying-induced shrinkage stresses [10]. The film-forming outcome is therefore specific to the applied conditions, and the non-film-forming polymers might form films in the presence of plasticizers, in combination with other film-forming polymers or under modified casting and drying conditions. Since the present study aimed to compare the polymers in their pure, drug- and excipient-free state, such formulation variables were not introduced, and film characterization was restricted to the four film-forming polymers.

### 2.3. Apparent Viscosity

**Figure 2:**
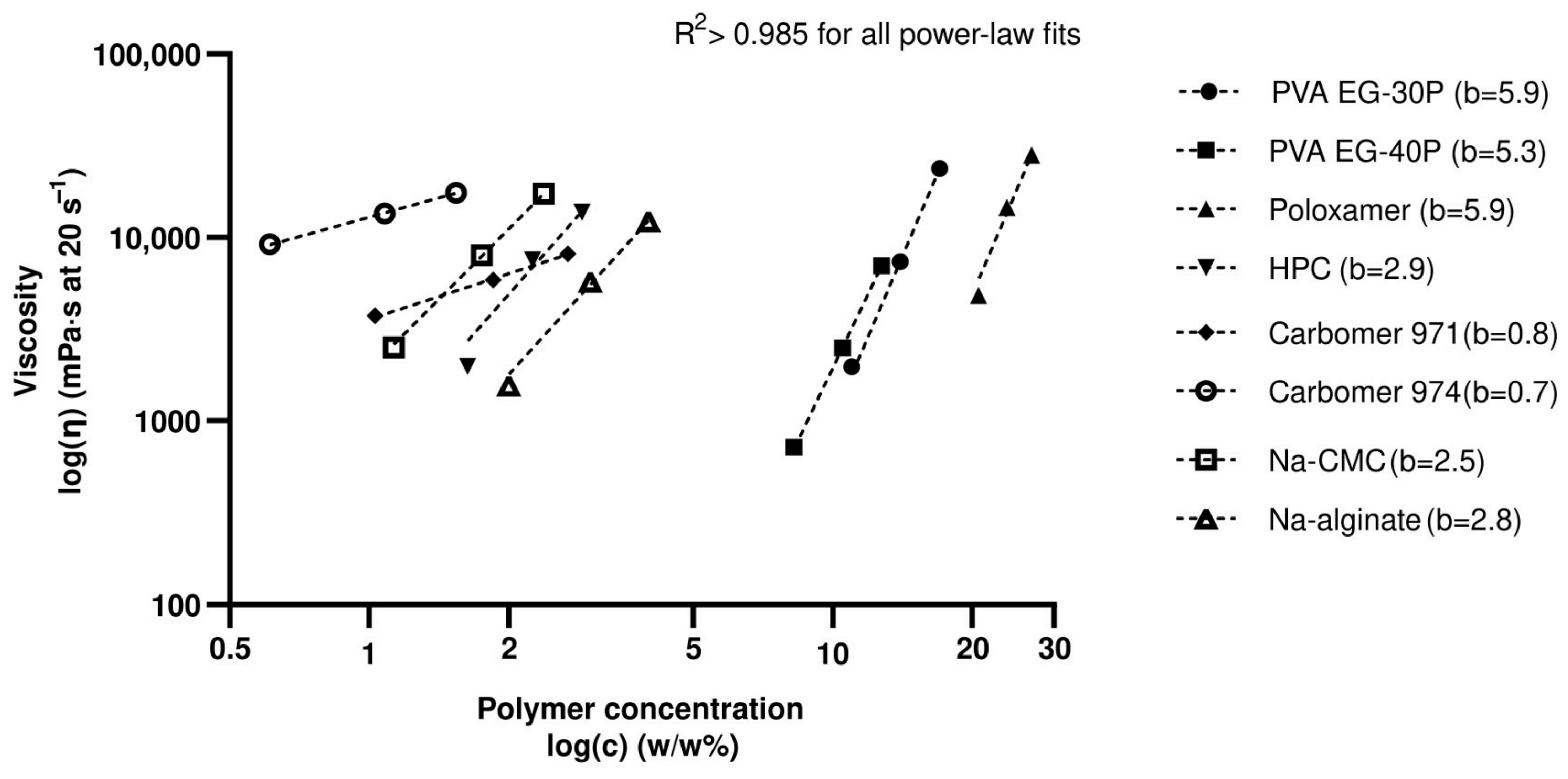
Apparent viscosity data of polymeric systems measured at c1, c2, and c3 concentrations, at a shear rate of 20 s^−1^. Data are presented as mean (*n* = 3, SD < 5%) and are plotted on logarithmic axes. The concentration-viscosity slopes (b) were calculated as b = Δlog(η)/Δlog(c).

Apparent viscosity was measured for all eight polymers at three concentrations (c1, c2, and c3) at a fixed shear rate of 20 s^−1^. The obtained viscosity values were used as comparative screening parameters under standardized measurement conditions. The results are presented in Figure 2. The data are displayed on logarithmic axes to better visualize the concentration-dependent trends across the wide viscosity range covered by the polymer set.

The results showed that the apparent viscosity depended on both the polymer type and concentration, and increased with increasing polymer concentration in all investigated systems. This behavior reflects polymer-specific differences in molecular weight, chain architecture (chain length and degree of branching), ionic character, and intermolecular interactions. As the polymer concentration increases, polymer chains overlap to a greater extent, promoting the formation of entanglements, thereby increasing viscosity [25]. Depending on the polymer type, the hydrogen bonding, electrostatic repulsion, chain entanglement, micellar association, and crosslinking of polymer chains may contribute to the observed concentration-dependent behavior [26].

Among the investigated formulations, Poloxamer exhibited the highest apparent viscosity values, whereas Carbomer 974 showed a high apparent viscosity already at low polymer concentrations. For Poloxamer, this behavior is consistent with its amphiphilic character and the formation of a supramolecular, self-assembled micellar network, which is concentration- and temperature-dependent [15,16]. In contrast, the high apparent viscosity of Carbomer 974, even at substantially lower polymer concentrations, can be attributed to its high molecular weight, and its crosslinked poly(acrylic acid) structure [17]. Because Carbomer viscosity is strongly dependent on the degree of ionization and neutralization, all Carbomer formulations were evaluated within a controlled pH range of 7.0 ± 0.2 to minimize any pH-related variability [17].

PVA EG-30P (b = 5.9) and Poloxamer (b = 5.9) exhibited the steepest concentration-viscosity slopes, followed by PVA EG-40P (b = 5.3), indicating a strong concentration dependence of the apparent viscosity values. In contrast, both Carbomer grades were found to be the least sensitive to concentration changes within their examined concentration ranges (Carbomer 971: b = 0.8, Carbomer 974: b = 0.7). In the case of Carbomers, the apparent viscosity increase is associated not only with polymer concentration but also with network swelling and the ionization state of the carboxyl groups. Na-CMC (b = 2.5), HPC (b = 2.9), and Na-alginate (b = 2.8) exhibited more moderate slopes compared with the PVA grades and Poloxamer, suggesting that the concentration dependence is less pronounced.

### 2.4. Thin-Film Thickness

The thickness of the PVA EG-30P, PVA EG-40P, HPC, and Na-alginate thin films increased with polymer concentration for all formulations. The PVA-based systems produced the thickest films, with PVA EG-30P increasing from 162.2 ± 5.7 µm at c1 to 334.6 ± 7.6 µm at c3, while PVA EG-40P increased from 156.6 ± 3.0 µm at c1 to 226.8 ± 8.6 µm at c3. In contrast, HPC and Na-alginate formed much thinner matrices, with thickness values increasing from 50.0 ± 2.4 µm to 93.2 ± 2.6 µm and from 46.2 ± 1.9 µm to 101.0 ± 4.8 µm between c1 and c3, respectively. These data reflect that the polymer type and concentration allow the preparation of films with a different thickness under identical processing conditions. Because all gels were cast into templates of identical depth and dried under identical conditions, the dry film thickness depends on the amount of polymer deposited per unit area and on how the gel layer levels and shrinks during drying, both of which are influenced by the viscosity of the cast gel. The two PVA grades have the same degree of hydrolysis (86.5–89.0 mol%) but differ in their degree of polymerization, as reflected by the typical viscosities of their 4% aqueous solutions at 20 °C (approximately 30 and 43 mm^2^/s for PVA EG-30P and PVA EG-40P, respectively) [27]. Owing to its higher molecular weight, PVA EG-40P reached the upper limit of its gelling range at a lower concentration than PVA EG-30P (15.00 vs. 20.00 *w*/*w*%; Section 3.2). Consequently, the c1–c3 levels of PVA EG-30P corresponded to 1.33-fold higher polymer contents (11.00–17.00% vs. 8.25–12.75% *w*/*w*), and its gels were approximately three times more viscous than the PVA EG-40P gels at the same concentration level (approximately 2.4 × 10^4^ vs. 7 × 10^3^ mPa·s at c3; Figure 2). From c1 to c3 concentrations, the polymer concentration increased 1.55-fold, whereas the thickness of the PVA EG-30P films increased 2.1-fold. This increase in film thickness was accompanied by a steep increase in gel viscosity (b = 5.9). Highly viscous gels are expected to level less readily in the template and to solidify at an earlier stage of drying, which may result in thicker films. Accordingly, the thickness difference between the two grades was small at c1 (162.2 ± 5.7 vs. 156.6 ± 3.0 µm), where both gels had comparatively low viscosities, and became pronounced at c3 (334.6 ± 7.6 vs. 226.8 ± 8.6 µm). The differences in dry film thickness between the two grades are therefore attributed primarily to their differences in molecular weight and the associated polymer concentrations and viscosities of the cast gels, rather than to differences in chemical composition.

### 2.5. Loss on Drying

**Figure 3:**
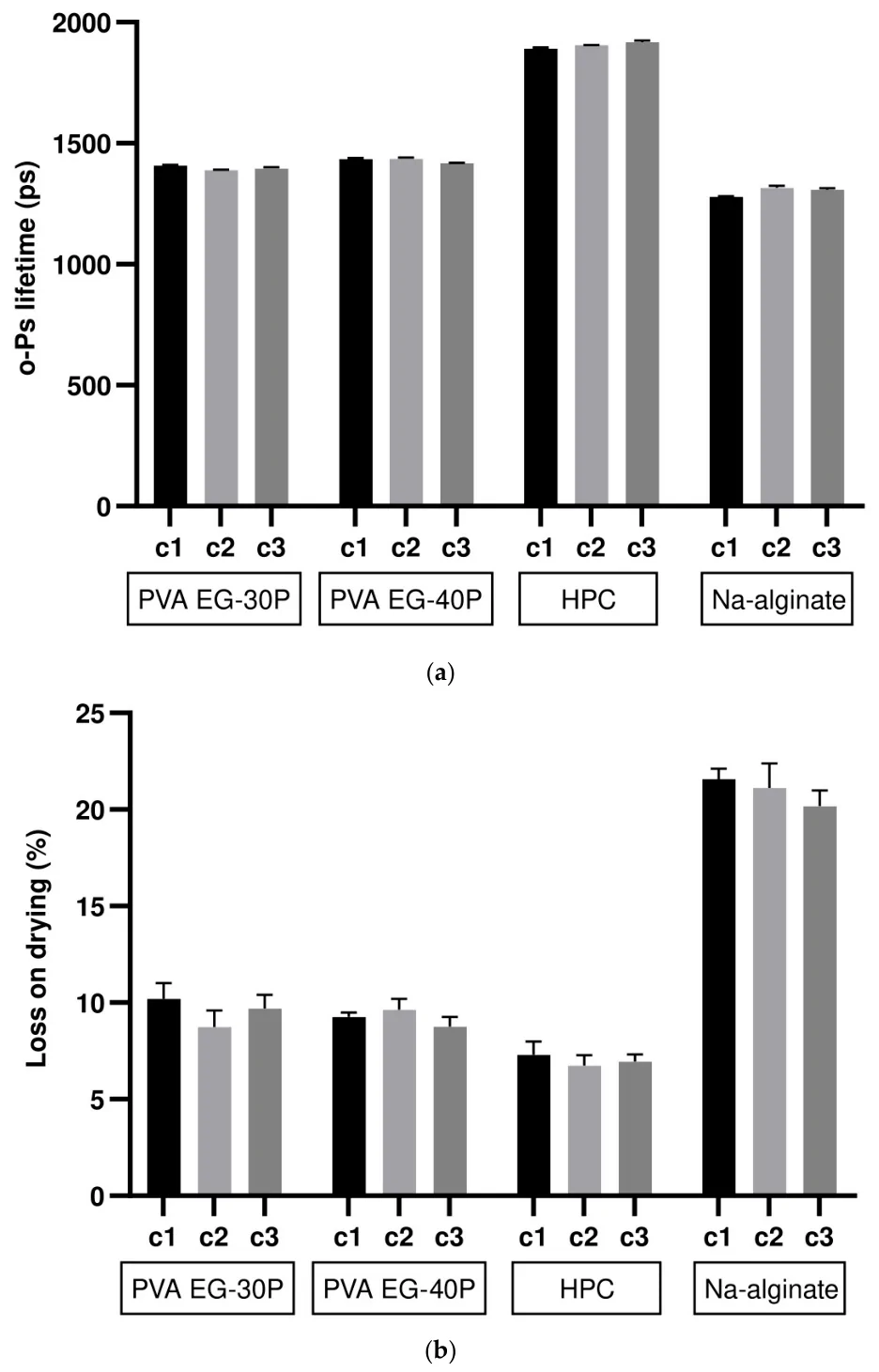
(**a**) Ortho-positronium (o-Ps) lifetime (τ_3_, ps) and (**b**) loss on drying (LOD, %) of PVA EG-30P, PVA EG-40P, HPC, and Na-alginate thin films prepared at the c1, c2, and c3 concentration levels (*w*/*w*%). Data are presented as means ± SD (*n* = 3 for the PALS data and *n* = 5 for the LOD data).

The residual water content of the polymer films was evaluated at three polymer concentration levels (c1, c2, and c3) by LOD measurement. The data are presented together with the PALS analysis results in Figure 3 and Table 3.

Na-alginate films demonstrated the highest LOD values exceeding 20%, whereas both PVA grades and HPC exhibited lower, moderate values ranging from 6.7 to 10.2%. The LOD values remained relatively consistent across concentration levels, indicating stable residual moisture content within each polymer system. Since LOD expresses the water content as a mass fraction of the film and all films were dried under identical, controlled conditions (25 °C, 60% RH), the residual moisture content reflects the amount of water retained in the films at sorption equilibrium with the drying atmosphere. The equilibrium water content is governed by the intrinsic water sorption isotherm of the given polymer, rather than the polymer concentration of the starting gel. The initial polymer concentration affects the amount of water to be removed during drying and the resulting film thickness, but does not directly determine the equilibrium moisture content of the dried film [11,28]. The results are consistent with the ability of polymers to bind and retain water in the film matrices, which are influenced by their chemical structure, ionic character and accessibility of ionizable or hydrophilic functional groups [11,28,29].

### 2.6. Positron Annihilation Lifetime Spectroscopy

PALS was performed on polymeric thin films to characterize their microstructure, and free volume properties, and to analyze the relationship between PALS-derived descriptors and LOD values across the examined concentration levels. Figure 3 and Table 3 report the o-Ps lifetime component (τ_3_), its relative intensity (I_3_), the estimated mean free volume hole radius (R), and the corresponding free volume hole volume (V_h_), and LOD values.

The derived PALS parameters represent nanoscale descriptors of local free volume and should not be interpreted as direct measures of morphological pore size or porosity.

Based on the PALS measurements, the studied polymers exhibited distinct microstructural characteristics and LOD values. An inverse relationship was observed between the PALS-derived free volume parameters and residual water content at the polymer level (Section 2.8). The differences between the results can be attributed to different chemical structures, the presence of side chains, the rigidity of polymer chains, the density of crosslinks, and the degree of crystallinity. Rigid chains or bulky side groups tend to increase the free volume, while a high degree of crystallinity or extensive crosslinking reduces it. Functional groups and retained water, which is highly polar, may also modulate the local electron density and affect the o-Ps lifetime [5,6]. Consequently, the structural differences influence the size and distribution of free volume in the polymer matrix and may affect the local electron environment probed by positronium.

The two PVA grades exhibited similar mean o-Ps lifetimes (τ_3_ = 1.39–1.44 ns) and intensities (I_3_ = 22.7–23.8%), suggesting comparable free volume environments. The corresponding free volume hole volumes (V_h_) ranged from 45.0 to 48.5 Å^3^, while LOD values remained moderate (8.7–10.2%). The results are consistent with the semicrystalline nature of PVA matrices, in which intra- and intermolecular hydrogen bonding may restrict chain mobility and influence the size and distribution of free volume cavities [12,30].

HPC films exhibited the longest mean o-Ps lifetimes (τ_3_ = 1.89–1.92 ns), intermediate o-Ps intensities (I_3_ = 19.1–21.0%), and the largest V_h_ values (86.7–89.5 Å^3^). These results indicate comparatively larger local free volume holes in the HPC matrix, whereas the o-Ps formation probability, reflected by I_3_, was intermediate between those of the PVA and Na-alginate films. HPC also showed the lowest LOD values ranging from 6.7 to 7.3%, indicating that a larger local free volume in the dry state was not associated with a higher residual water content.

Na-alginate exhibited the shortest mean o-Ps lifetimes (τ_3_ = 1.28–1.32 ns), the lowest o-Ps intensities (I_3_ = 14.0–15.9%), and the smallest V_h_ values (36.9–39.6 Å^3^) among the examined films. These findings indicate that the matrix environment is less favorable for o-Ps formation and is characterized by smaller effective local free volume holes. Data may also reflect that the free volume cavities are partially filled with retained water. This behavior may be related to the strong polymer–water interaction, due to the ionizable carboxylate and hydroxyl groups, which promote water retention within the matrix [8].

Overall, our results demonstrate that PALS distinguished the polymer matrices at a nanoscale level, according to their local free volume environments, and provided complementary information to the LOD measurements. However, the proposed mechanistic interpretation should be supported with complementary analysis of morphology, molecular mobility, and crystallinity.

### 2.7. Swelling Behavior

**Figure 4:**
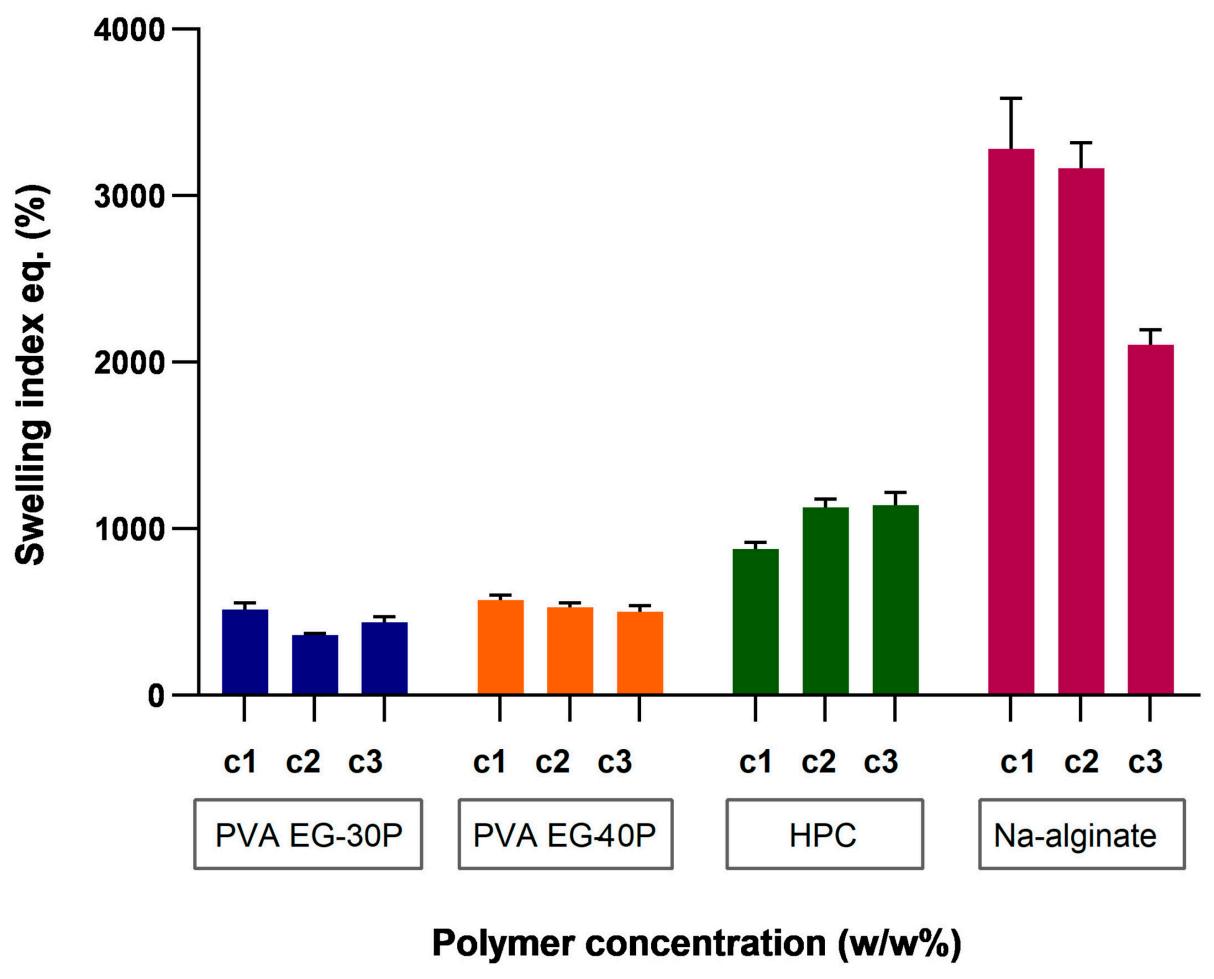
Equilibrium swelling index (SI_eq_) of the polymeric inserts measured at three polymer concentrations (c1, c2, and c3). Bars represent the mean ± SD (*n* = 3). The relative standard deviation was below 10% for all formulations.

The equilibrium swelling index (SI_eq_) was determined at c1, c2, and c3 concentration levels after a stable hydrated mass was reached. The results are presented in Figure 4.

The swelling behavior depended strongly on polymer type, and differences were also observed between the concentration levels within each polymer system. SI_eq_ ranged from 360.8 ± 10.0% to 3282.5 ± 301.8%. Two-way ANOVA revealed the significant effects of polymer type [*F*(3,24) = 973.9, *p* < 0.0001] and polymer concentration [*F*(2,24) = 22.75, *p* < 0.0001] on SI_eq_. A significant polymer type × polymer concentration interaction was also observed [*F*(6,24) = 31.07, *p* < 0.0001], indicating that the effect of polymer concentration on swelling depended on the polymer type.

Na-alginate exhibited the highest SI_eq_ values, reaching 3282.5 ± 301.8%, 3163.5 ± 154.2%, and 2105.6 ± 90.1% for c1, c2, and c3, respectively. The pronounced swelling capacity may be attributed to the hydrophilic and ionizable carboxylate groups of Na-alginate, which promote electrostatic repulsion, water uptake, and matrix expansion in PBS [31,32]. Tukey’s multiple-comparisons test showed no significant difference between c1 and c2 (*p* = 0.3816). However, the SI_eq_ of c3 was significantly lower than those of c1 and c2 (both adjusted *p* < 0.0001). This finding suggests that a higher alginate concentration may form a more compact and highly entangled system, thereby limiting PBS diffusion into the matrix and restricting network expansion [32].

HPC-based inserts showed intermediate SI_eq_ values, ranging from 877.1 ± 41.4% to 1140.3 ± 79.6%. In contrast to the PVA and Na-alginate systems, SI_eq_ increased with increasing HPC concentration. This behavior may be associated with the hydrophilic hydroxyl and hydroxypropyl groups of HPC, which facilitate hydrogen bonding interactions with water molecules and promote water uptake [33,34]. The SI_eq_ values of c2 and c3 were significantly higher than that of c1 (adjusted *p* = 0.0221 and *p* = 0.0168, respectively). A plateau was observed between c2 and c3, with no significant difference between the formulations (adjusted *p* = 0.9921). This plateau may indicate that an increased polymer concentration, chain entanglement, and matrix densification restrict the further chain mobility and expansion of the hydrated network [35].

PVA-based inserts exhibited the lowest SI_eq_ values, ranging from 360.8 ± 10.0% to 514.7 ± 40.6% for PVA EG-30P and from 503.0 ± 35.5% to 570.4 ± 32.5% for PVA EG-40P. Both PVA grades showed relatively low swelling capacities, with lower SI_eq_ values at c2 and c3 than at c1. This limited swelling may be related to their stronger intermolecular interactions and the semicrystalline structure of PVA, which restrict water penetration and lead to a more controlled matrix expansion [36,37]. The small, statistically non-significant differences between the two PVA grades may be associated with their different molecular weights, which can affect chain packing and crystallinity [36].

**Figure 5:**
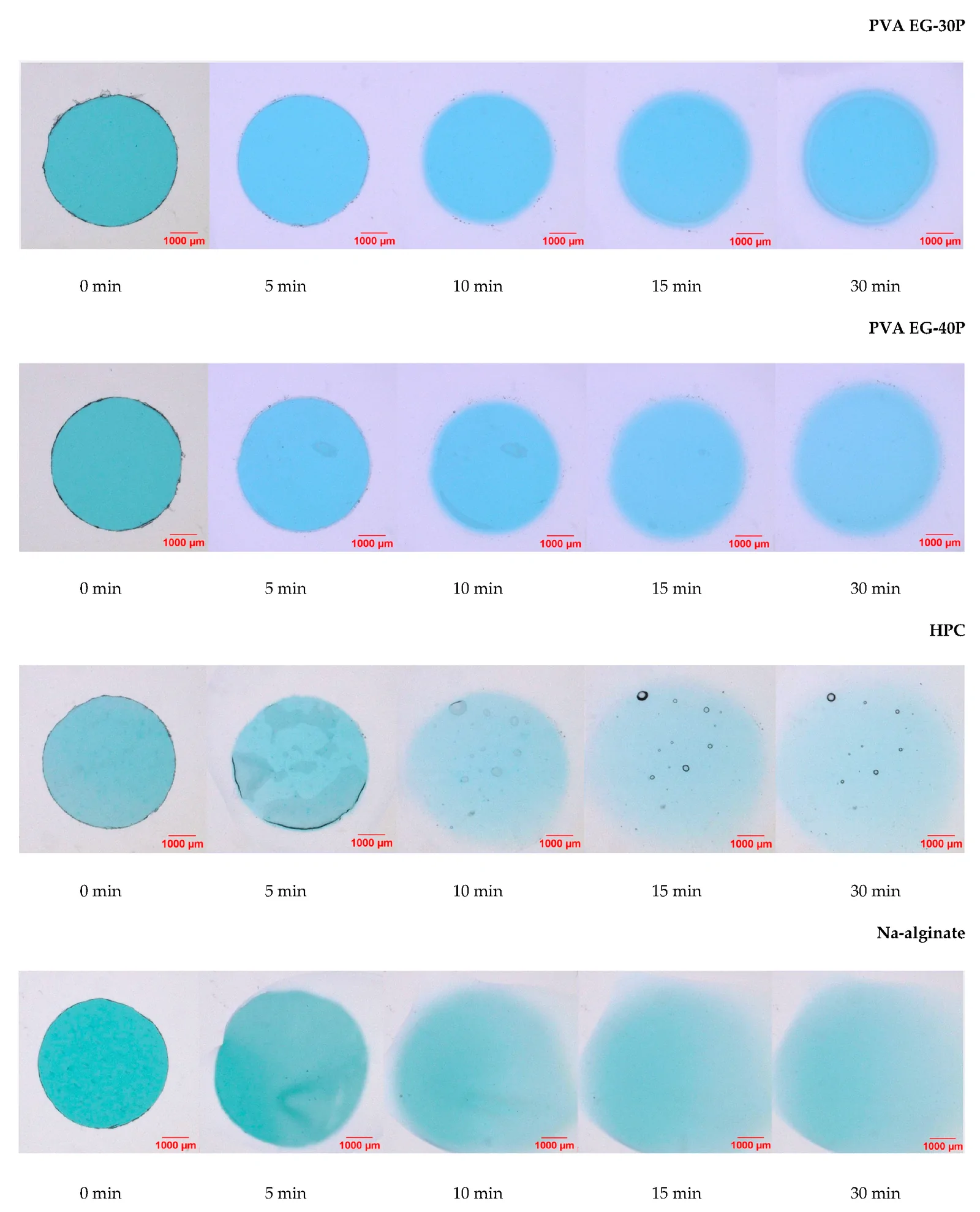
Hydration-associated diameter changes of polymer films prepared at the c2 concentration level. Images show the dry films (0 min) and the swelling of the films 5, 10, 15, and 30 min after the application of 100 µL of PBS (pH 7.4). Scale bar: 1000 µm.

To complement the SI_eq_ results, the hydration behavior of polymeric inserts prepared at c2 concentration level was evaluated by monitoring changes in the diameter over a 30 min period. While SI_eq_ quantifies mass-based swelling after the inserts reached a stable hydrated mass, changes in the insert diameter illustrated the time-dependent, lateral dimensional response during hydration. Figure 5 shows representative images of the inserts in dry state and during swelling at 0, 5, 10, 15, and 30 min, and the corresponding quantitative data are summarized in Table 4.

The time-resolved diameter measurements were consistent with the SI_eq_ analysis results. The formulations with higher SI_eq_ values also exhibited a greater dimensional expansion at the examined c2 concentration level. PVA EG-30P and PVA EG-40P exhibited moderate lateral expansion after 30 min (11.9% and 12.1%, respectively). In contrast, HPC and Na-alginate displayed more pronounced total diameter increases of 29.5% and 30.7%, respectively.

These findings highlight that, although both Na-alginate and HPC inserts exhibited extensive swelling, this is governed by distinct microstructural properties and mechanisms: Na-alginate swelling is governed by ionic, strongly water-binding groups, whereas HPC displays rapid hydration, which may be governed by an open, loosely packed, hydrogen-bonding matrix [32,33,34]. In contrast, a more gradual and limited swelling of both PVA matrices was observed, consistent with their lower SI_eq_ values. This may be associated with their semicrystalline network structure and intermolecular hydrogen bonding, which can limit chain mobility and matrix expansion [36,37]. Similar diameter changes were observed for both PVA grades at c2 concentration.

### 2.8. Relationship Between Free Volume and Hydration-Related Properties

**Figure 6:**
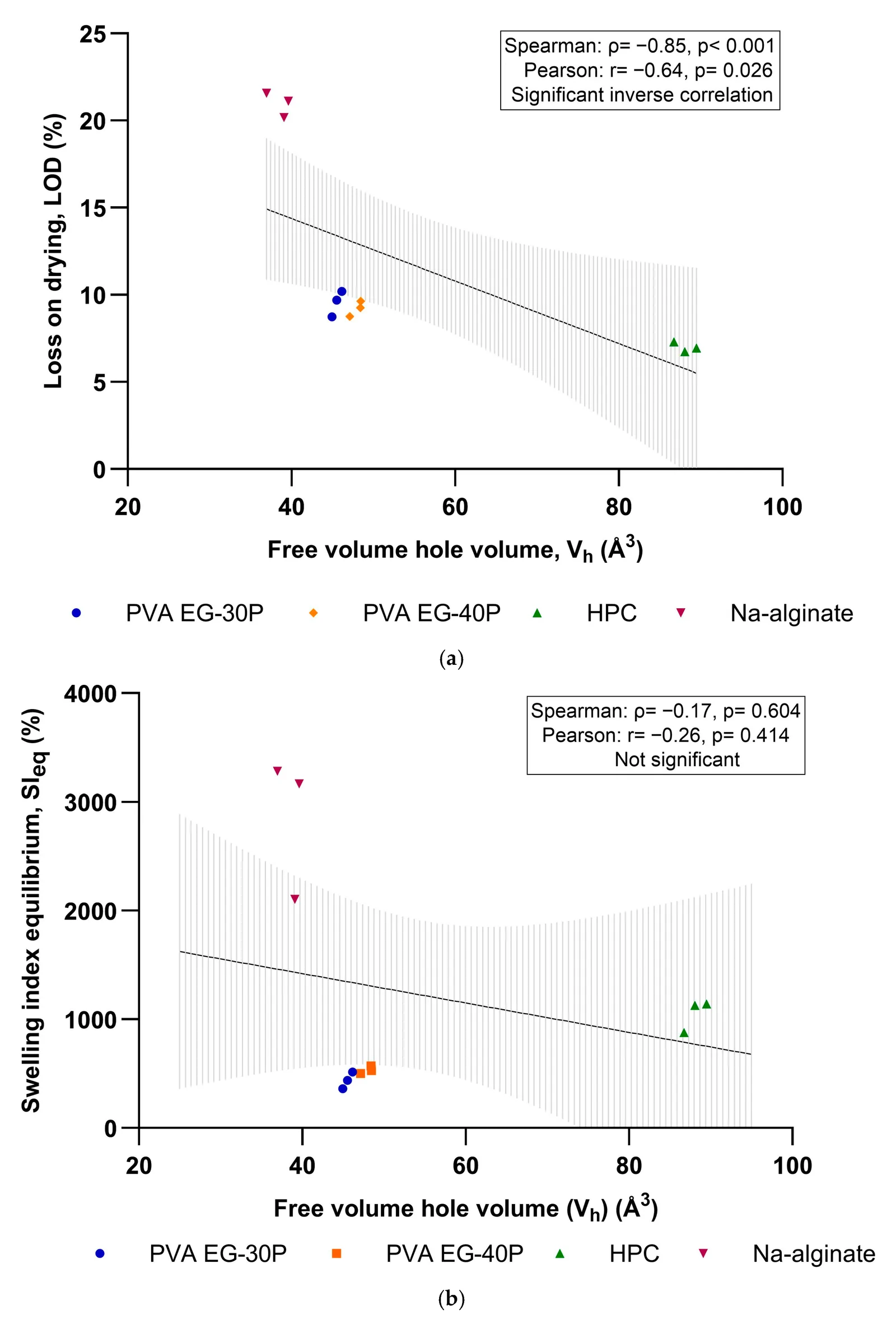
Correlations between PALS-derived free volume hole volume (V_h_, Å^3^) and (**a**) loss on drying (LOD, %) and (**b**) equilibrium swelling index (SI_eq_, %) of polymeric film formulations. Each point represents paired mean V_h_ values and mean LOD values or mean SI_eq_ values for one concentration level (c1–c3) of a given polymer. PVA EG-30P, PVA EG-40P, HPC, and Na-alginate formulations are distinguished by symbol and color. The dashed lines represent the pooled ordinary least squares linear fits, and the shaded areas indicate the 95% confidence bands. Across all 12 concentration-level observations, V_h_ was inversely correlated with LOD (Spearman ρ = −0.85, *p* = 0.0009; Pearson *r* = −0.64, *p* = 0.0258). No statistically significant correlation was observed between V_h_ and SI_eq_ (Spearman ρ = −0.17, *p* = 0.6039; Pearson *r* = −0.26, *p* = 0.4142).

To evaluate whether the free volume of the air-dried films is related to their hydration behavior, the mean V_h_ values were correlated with the corresponding mean LOD and SI_eq_ values of each polymer at each concentration level (12 polymer–concentration combinations; Figure 6). V_h_ was inversely correlated with LOD (Spearman ρ = −0.85, *p* = 0.0009; Pearson *r* = −0.64, *p* = 0.0258; Figure 6a). This relationship was determined primarily by the differences between the polymer types: Na-alginate films combined the smallest free volume holes with the highest residual water content, HPC films showed the opposite combination, and the two PVA grades occupied an intermediate position. Within each polymer system, variation across concentration levels of a given polymer was small (Table 3).

Thus, the overall correlation primarily reflects differences among polymer types rather than concentration-dependent differences within each polymer system (Section 2.9). The inverse relationship is consistent with the stronger water retention of the ionic Na-alginate matrix, in which the retained water may partially occupy the free volume cavities and modify the local environment probed by o-Ps [5,6,8,30].

In contrast, no statistically significant correlation was found between V_h_ and SI_eq_ (Spearman ρ = −0.17, *p* = 0.6039; Pearson *r* = −0.26, *p* = 0.4142; Figure 6b). HPC and Na-alginate, which represent the two extremes of V_h_ (86.7–89.5 Å^3^ and 36.9–39.6 Å^3^, respectively), both showed considerably higher SI_eq_ values and diameter increases than the PVA films, which had intermediate V_h_ values (Figure 4 and Figure 5, and Table 4). Moreover, the amount of medium taken up at equilibrium (SI_eq_ = 360.8–3282.5%) far exceeds the free volume of the air-dried matrices. Therefore, swelling is not limited by the pre-existing free volume, but by polymer-solvent interactions and the elastic restraint of the hydrated network, which are governed by polymer-specific features, such as the ionized carboxylate groups of Na-alginate, the hydrophilic, loosely packed hydroxypropyl-substituted chains of HPC, and the crystallite-reinforced, hydrogen-bonded network of PVA [26,32,33,34,36,37]. Accordingly, the PALS-derived free volume descriptors of the air-dried films are best regarded as complementary microstructural parameters that are linked to the residual water content, rather than as stand-alone predictors of the swelling behavior.

### 2.9. Limitations

The main findings of this study, i.e., the polymer-specific differences in the PALS-derived free volume descriptors, the inverse relationship between V_h_ and LOD, and the absence of a significant relationship between V_h_ and SI_eq_, are directly supported by the measured data. In contrast, the molecular-level mechanisms proposed to explain these findings (e.g., the contributions of crystallinity, interchain hydrogen bonding, chain mobility, and water-filled cavities) are based on the PALS, LOD, and swelling measurement data interpreted in the light of the literature, and should therefore be regarded as hypotheses. The complementary characterization of morphology, crystallinity, and molecular mobility (e.g., by X-ray diffraction, differential scanning calorimetry, dynamic mechanical analysis, or solid-state NMR) would be required to substantiate them. Moreover, the correlation analysis (Section 2.8) is based on 12 polymer–concentration combinations of only 4 polymers; therefore, the observed relationships mainly reflect between-polymer differences and should be verified on a broader set of polymers. In addition, the viscosity measurements were performed at a single shear rate and therefore serve as a comparative screening tool under standardized conditions, and not as a comprehensive rheological characterization. Finally, the films investigated in this study are API-free and contain no formulation-specific excipients. Consequently, the influence of APIs and excipients on the local free volume and hydration behavior remains to be established.

## 3. Materials and Methods

### 3.1. Materials

Poly(vinyl alcohol) (PVA, Gohsenol EG-30P, Gohsenol EG-40P) was provided by HARKE Pharma GmbH (Mülheim an der Ruhr, Germany). Poloxamer 407 (Poloxamer, Kolliphor P407) was purchased from BASF SE (Ludwigshafen, Germany). Carbomer 971 and Carbomer 974 (Carbopol 971, Carbopol 974) were supplied by Lubrizol Corporation (Wickliffe, OH, USA). Hydroxypropyl cellulose (HPC, Klucel HF) and sodium carboxymethylcellulose (Na-CMC, Aqualon CMC 7H4F PH) were provided by Ashland Inc. (Wilmington, DE, USA). Sodium alginate (Na-alginate, Alginate PH 155) was obtained from JRS Pharma GmbH & Co. KG (Rosenberg, Germany). Lynbond E2 PVA sponge was purchased from Suntec Solution Hungary Kft. (Budapest, Hungary).

### 3.2. Preparation of Hydrogels and Polymeric Thin Films

All polymers were prepared at three concentration levels (c1, c2, and c3, *w*/*w*%, Table 5). These levels represent evenly distributed points within the gelling concentration range of each polymer. Minimum and maximum gelling limits were empirically determined.

The PVA grades were dispersed in purified water, heated at 80–90 °C for 30 min, and cooled under continuous stirring. Poloxamer was dissolved in cold water (≤4 °C), and allowed to warm to room temperature after dissolution. Carbomer 971 and 974 grades were dispersed, hydrated, and neutralized to pH 7.0 using 10% (*w*/*w*) NaOH solution. Na-CMC, HPC, and Na-alginate were gradually dispersed at room temperature under continuous stirring. Samples were deaerated using an ultrasonic bath for 10–30 min at 25 °C and allowed to rest for at least 12 h.

The hydrogels were cast into 300 μm-deep silicone templates and distributed evenly. The films were dried under controlled conditions at 25 °C and 60% RH and analyzed within two weeks of preparation.

### 3.3. Viscosity Measurement

Gel preparation was carried out for each polymer in three concentrations (c1, c2, and c3, *w*/*w*%) following the established protocol. Apparent viscosity was measured at a fixed shear rate of 20 s^−1^ using Lamy First Prodig CP-1000 plate-plate rheometer (Lamy Rheology Instruments, Champagne-au-Mont-d’Or, France; *n* = 3) in a controlled environment at 22 °C and 40% RH.

The viscosity measurements were performed at a single shear rate and were used for comparative screening of the polymer formulations under standardized conditions. Therefore, the data do not represent a comprehensive rheological characterization.

LOD, PALS, and swelling tests were performed only on polymers selected as film-forming materials for the polymer film matrix systems.

### 3.4. Thin Film Thickness

Film thickness was measured using a digital micrometer (Yato, YT-72305; TOYA S.A., Wrocław, Poland; resolution: 0.001 mm, precision: ±0.002 mm), at five different locations on each thin film, and the average thickness was calculated (*n* = 5).

### 3.5. Loss on Drying

PVA EG-30P, PVA EG-40P, HPC, and Na-alginate thin films were prepared at c1, c2, and c3 concentrations (*w*/*w*%). LOD was determined using a Scaltec SMO 01 halogen moisture analyzer (Scaltec Instruments GmbH, Göttingen, Germany). Film samples (0.5 g each, *n* = 5) were analyzed at a set temperature of 160 °C. Since no volatile solvents or plasticizers were used in the formulations other than purified water, the LOD results were attributed primarily to the residual water content. Loss on drying (LOD) (%) was calculated as follows:

(1) $$\mathrm{LOD}\;(\%)\;=\;\frac{\mathrm{Weight loss}\;(g)}{\mathrm{Total sample weight}\;(g)}\times 100$$

### 3.6. Positron Annihilation Lifetime Spectroscopy

PVA EG-30P, PVA EG-40P, HPC, and Na-alginate thin films were prepared at c1, c2, and c3 concentrations (*w*/*w*%). The PALS measurements were performed using a conventional fast-fast coincidence setup equipped with BaF_2_ scintillation crystals, XP2020Q photomultiplier tubes (Koninklijke Philips N.V., Eindhoven, The Netherlands), and ORTEC electronics (AMETEK ORTEC, Oak Ridge, TN, USA). The positron source was prepared from carrier-free ^22^NaCl (activity ~10^5^ Bq) sealed between two thin Kapton foils (~7 μm thickness). Three parallel measurements were performed for each composition, and the resulting spectra were analyzed using the RESOLUTION routine of the PATFIT-88/PALSfit program package [38,39]. Positron lifetimes, o-Ps lifetimes (τ_3_), and their respective relative intensities (I_3_) were determined by this code. The Tao–Eldrup model was applied to calculate the free volume hole radius R and the corresponding free volume hole volume V_h_ = 4/3πR^3^ [3,4].

### 3.7. Swelling Behavior

Swelling behavior was evaluated by determining the equilibrium swelling index and monitoring dimensional changes using digital microscopy. For swelling measurement preparation, two layers of PVA sponge (12 cm × 12 cm, 130 µm nominal pore size) were placed in the soaking chamber, saturated with 55 mL of phosphate-buffered saline solution (PBS, pH 7.4) and incubated at 37 °C. The inserts were separated from the PVA sponge using glass microfiber filter papers (Whatman glass microfiber filter, grade GF/C, 1.2 µm pore size) to prevent direct contact and adhesion of the inserts to the PVA sponge. The filters were pre-wetted for 15 min on the PVA sponge. Liquid retention by the glass microfiber filters was determined separately in blank experiments under identical conditions and was corrected in the calculations (by subtracting the blank support weights from the recorded insert-support weights).

For swelling index evaluation, circular inserts (Ø = 15 mm, *n* = 3) were initially weighed in dry state, then placed on the pre-wetted glass microfiber filters. The insert-support combinations were weighed every hour, until the inserts reached a constant weight. Equilibrium swelling was considered reached when the relative difference between two consecutive corrected insert weights was ≤5%.

The equilibrium swelling index (SI_eq_) (%) was calculated as follows [40]:

(2) $${\mathrm{SI}}_{\mathrm{eq}}\;(\%)\;=\;\frac{W_{\mathrm{eq}}-W_{0}}{W_{0}}\times 100$$

where W_eq_ is the corrected weight of the insert at equilibrium swelling and W_0_ is the initial dry weight of the insert.

A Keyence VHX-970F digital microscope (Keyence Corporation, Osaka, Japan) was used to visualize the swelling process of the inserts. Images of the circular inserts (Ø = 5 mm, *n* = 3) were captured in the dry state (0 min) and at 5, 10, 15, and 30 min after the application of 100 µL PBS directly dropped on the insert surfaces. The insert diameter was measured at each of these time points to quantify the extent of radial expansion. The polymer films were stained with a blue dye prior to hydration for visualization. The total insert diameter increase after 30 min of hydration was calculated as follows:

(3) $$\mathrm{Total}\;\mathrm{diameter}\;\mathrm{increase}\;(\%)\;=\;\frac{D_{30}-D_{0}}{D_{0}}\times 100$$

where D_0_ is the initial mean diameter of the dry insert and D_30_ is the mean diameter measured after 30 min of hydration.

### 3.8. Statistical Analysis

Statistical analysis was performed to evaluate the effects of polymer type, polymer concentration, and their interaction on the equilibrium swelling index using two-way analysis of variance (ANOVA). Tukey’s multiple-comparisons test was used for post hoc pairwise comparisons. Differences among concentration levels within each polymer type, and differences at each concentration level among polymer types were assessed. Correlations between the mean V_h_ values and the corresponding mean LOD and SI_eq_ values (12 polymer–concentration combinations) were evaluated by Spearman’s rank correlation and Pearson’s correlation analyses, and pooled ordinary least squares linear fits with 95% confidence bands were used for visualization (Figure 6). Differences were considered statistically significant at *p* < 0.05. GraphPad Prism software, version 8.0 (GraphPad Software, San Diego, CA, USA), was used for the statistical analysis.

## 4. Conclusions

This study demonstrates the applicability of PALS for the microstructural characterization and differentiation of pharmaceutical polymer thin films. PALS-derived free volume parameters were evaluated together with loss on drying, equilibrium swelling, and time-dependent dimensional changes to compare the local free volume environment with hydration-related characteristics. This combined analysis enabled the comparison of nanoscale free volume descriptors with macroscopic hydration-related properties of the polymeric matrices, and provided a basis for the interpretation of potential underlying molecular mechanisms.

Under the applied preparation conditions, only four of the eight screened polymers (PVA EG-30P, PVA EG-40P, HPC, and Na-alginate) formed continuous films. PALS clearly differentiated between the HPC, Na-alginate, and PVA matrices, whereas the two PVA grades showed comparable values and the polymer concentration of the casting gel had only a minor effect on the free volume descriptors: the HPC films contained the largest and the Na-alginate films the smallest free volume holes. The free volume hole volume of the films was inversely related to their residual moisture content but not to their swelling capacity. Consequently, the PALS-derived free volume descriptors of air-dried films are suitable for discriminating film-forming pharmaceutical polymers and for complementing residual moisture data, but they cannot be used as stand-alone predictors of the swelling capacity of the films.

These results support the use of PALS as a complementary microstructural tool in the QbD-based selection of film-forming polymers. Further studies on a broader set of polymers and on API- and excipient-containing films, combining PALS with the characterization of morphology, crystallinity, and molecular mobility, are needed to confirm these relationships and to substantiate the proposed mechanistic interpretations.

## Figures and Tables

**Table 1:** FMEA-based risk assessment of the critical material attributes (CMAs) and critical process parameters (CPPs) affecting the critical quality attributes (CQAs) of API-free polymeric thin films.

Factor (CMAs, CPPs)	Linked Thin Film Property (CQA) *					Risk Assessment **				
	Thickness	Moisture Content	Mechanical Properties	Microstructure	Swelling Behavior	S	O	D	RPN	Qualitative Risk
Polymer material attributes										
Molecular weight	✓		✓	✓	✓	4	3	3	36	High
Concentration	✓	✓	✓	✓	✓	5	4	2	40	High
Ionic nature		✓	✓	✓	✓	4	3	3	36	High
Gel system attributes										
pH					✓	4	2	2	16	Low
Viscosity	✓	✓	✓	✓	✓	5	4	2	40	High
Viscoelastic behavior			✓	✓	✓	4	3	3	36	High
Gel preparation										
Temperature				✓		4	3	2	24	Medium
Mixing				✓		3	4	2	24	Medium
Time				✓		3	3	3	27	Medium
Thin film preparation										
Temperature	✓	✓	✓	✓	✓	5	3	2	30	High
Humidity	✓	✓	✓	✓	✓	4	4	2	32	High
Time	✓	✓	✓	✓	✓	4	3	3	36	High

* ‘✓’ sign indicates that the CMA or CPP factor has relevant impact on the corresponding CQA. Blank cells indicate negligible impact. ** Risk assessment was performed considering the overall impact of each CMA/CPP on all linked CQAs (S, severity; O, occurrence; D, detectability; RPN, risk priority number).

**Table 2:** Chemical classification and film-forming outcomes of the polymers evaluated under the applied preparation, casting, and drying conditions.

Polymer	Film-Forming Capacity	Ionic Nature	Origin	Relevant Structural Features
PVA EG-30P	Continuous film	Non-ionic	Synthetic	Semicrystalline vinyl polymer rich in hydroxyl groups [11,12]
PVA EG-40P	Continuous film	Non-ionic	Synthetic	Semicrystalline vinyl polymer rich in hydroxyl groups [11,12]
HPC	Continuous film	Non-ionic	Semisynthetic	Cellulose ether with hydroxypropyl groups [13,14]
Na-alginate	Continuous film	Anionic	Naturally derived	Polysaccharide with carboxylate groups; ion-responsive gelling polymer [8,9]
Carbomer 971	No continuous film formed	Anionic	Synthetic	Crosslinked poly(acrylic acid) network [17]
Carbomer 974	No continuous film formed	Anionic	Synthetic	Crosslinked poly(acrylic acid) network [17]
Na-CMC	No continuous film formed	Anionic	Semisynthetic	Carboxymethyl-substituted cellulose ether [10]
Poloxamer	No continuous film formed	Non-ionic	Synthetic	Amphiphilic; PEO-PPO-PEO triblock copolymer; thermoresponsive gelling polymer [15,16]

**Table 3:** Ortho-positronium (o-Ps) lifetime (τ_3_, ps) and relative intensity (I_3_, %), estimated free volume hole parameters, and loss on drying (LOD, %) of the polymeric thin films at the c1, c2, and c3 concentration levels (*w*/*w*%). PALS and LOD data are presented as mean ± SD (*n* = 3 and *n* = 5, respectively).

Polymer	Conc. Level	Conc. (*w*/*w*%)	o-Ps Lifetime (τ_3_) (ps)	o-Ps Intensity (I_3_) (%)	Free Volume Hole Radius R (Å)	Free Volume Hole Volume V_h_ (Å^3^)	Loss on Drying (LOD) (%)
PVA EG-30P	c1	11.00	1407.0 ± 4.4	23.4 ± 0.1	2.225	46.14	10.2 ± 0.8
	c2	14.00	1389.3 ± 1.5	23.7 ± 0.1	2.206	44.97	8.7 ± 0.9
	c3	17.00	1396.0 ± 4.6	23.8 ± 0.1	2.215	45.52	9.7 ± 0.7
PVA EG-40P	c1	8.25	1434.3 ± 5.0	22.9 ± 0.1	2.261	48.42	9.3 ± 0.2
	c2	10.50	1435.0 ± 6.0	22.7 ± 0.2	2.262	48.48	9.6 ± 0.6
	c3	12.75	1417.7 ± 2.3	23.3 ± 0.1	2.241	47.14	8.8 ± 0.5
HPC	c1	1.63	1891.0 ± 5.3	19.1 ± 0.1	2.746	86.73	7.3 ± 0.7
	c2	2.25	1905.0 ± 1.0	20.8 ± 0.1	2.760	88.07	6.7 ± 0.5
	c3	2.88	1917.0 ± 7.0	21.0 ± 0.1	2.775	89.51	6.9 ± 0.4
Na-alginate	c1	2.00	1279.0 ± 2.6	14.0 ± 0.1	2.066	36.94	21.6 ± 0.5
	c2	3.00	1315.7 ± 9.0	15.4 ± 0.2	2.115	39.63	21.1 ± 1.3
	c3	4.00	1308.0 ± 5.6	15.9 ± 0.2	2.105	39.07	20.2 ± 0.8

**Table 4:** Diameter of the polymeric inserts prepared at the c2 concentration level during hydration in PBS (pH 7.4). Values are presented as mean ± SD (*n* = 3). The total diameter increase (%) was calculated relative to the diameter of the dry insert (0 min).

	PVA EG-30P	PVA EG-40P	HPC	Na-Alginate
Time point	Average diameter (mm) ± SD			
0 min	4.87 ± 0.07	4.89 ± 0.04	4.95 ± 0.24	5.86 ± 0.16
5 min	4.96 ± 0.08	5.17 ± 0.12	5.50 ± 0.56	6.49 ± 0.82
10 min	5.09 ± 0.12	5.32 ± 0.06	5.78 ± 0.36	6.62 ± 0.96
15 min	5.28 ± 0.34	5.25 ± 0.06	6.22 ± 0.28	7.21 ± 0.17
30 min	5.45 ± 0.36	5.48 ± 0.16	6.41 ± 0.12	7.66 ± 0.30
Total diameter increase (%)	11.9%	12.1%	29.5%	30.7%

**Table 5:** Concentration levels (c1, c2, and c3) (*w*/*w*%) and gelation ranges of the examined polymers.

Polymers	Minimum (*w*/*w*%)	c1 (*w*/*w*%)	c2 (*w*/*w*%)	c3 (*w*/*w*%)	Maximum (*w*/*w*%)
PVA EG-30P	8.00	11.00	14.00	17.00	20.00
PVA EG-40P	6.00	8.25	10.50	12.75	15.00
Poloxamer	17.50	20.63	23.75	26.88	30.00
HPC	1.00	1.63	2.25	2.88	3.50
Carbomer 971	0.20	1.03	1.85	2.68	3.50
Carbomer 974	0.15	0.61	1.08	1.54	2.00
Na-CMC	0.50	1.13	1.75	2.38	3.00
Na-alginate	1.00	2.00	3.00	4.00	5.00

## Data Availability

The data presented in this study are available upon request from the corresponding author.

## Abbreviations

The following abbreviations are used in this manuscript:

ANOVA	analysis of variance
API	active pharmaceutical ingredient
CMA	critical material attribute
CPP	critical process parameter
CQA	critical quality attribute
FMEA	failure mode and effects analysis
HPC	hydroxypropyl cellulose
LOD	loss on drying
Na-alginate	sodium alginate
Na-CMC	sodium carboxymethylcellulose
o-Ps	ortho-positronium
PALS	positron annihilation lifetime spectroscopy
PBS	phosphate-buffered saline
PEO	poly(ethylene oxide)
PPO	poly(propylene oxide)
PVA	poly(vinyl alcohol)
QbD	quality by design
RPN	risk priority number
SI_eq_	equilibrium swelling index
